# Clival Aneurysmal Bone Cyst With Cavernous Sinus Extension: A Diagnostic Challenge

**DOI:** 10.7759/cureus.115290

**Published:** 2026-08-27

**Authors:** Raphael Zimmermann, Érico Samuel Gomes Galvão da Trindade, Lucas Borges Alves, Williams Escalante Encinas, Allan Zimmermann

**Affiliations:** 1 Neurological Surgery, Hospital São Vicente de Paulo, Jundiaí, BRA

**Keywords:** aneurysmal bone cyst, case report, cavernous sinus, clivus, skull base, usp6

## Abstract

Aneurysmal bone cyst (ABC) is a benign but locally aggressive osseous neoplasm that predominantly affects children and young adults, with skull base involvement being uncommon. We report a 59-year-old woman with a progressively enlarging clival ABC extending into the sphenoid sinus and right cavernous sinus. She initially presented with right frontal headache and subsequently developed progressive diplopia, ptosis, ophthalmoplegia, and trigeminal sensory impairment. An initial endoscopic endonasal transsphenoidal biopsy was interpreted as a mucocele; however, continued clinical deterioration and radiological progression prompted repeat tissue sampling through a transcranial microsurgical approach. Histopathological and immunohistochemical findings, together with the detection of a USP6 gene rearrangement by fluorescence in situ hybridization, supported the diagnosis of primary ABC in the appropriate clinicopathological context. Complete surgical resection was not feasible because of extensive skull base and cavernous sinus involvement. The patient was referred to clinical oncology for consideration of denosumab; however, treatment was not recommended because the available supporting evidence was considered insufficient. The lesion continued to progress, with subsequent neurological deterioration, and the patient died approximately 10 months after the definitive diagnosis. This case illustrates the diagnostic challenges of ABC in an unusual anatomical location and age group and emphasizes the importance of clinicoradiological-pathological correlation, diagnostic reassessment when findings are discordant, and the appropriate use of ancillary molecular testing in selected cases.

## Introduction

Aneurysmal bone cyst (ABC) is a benign but locally aggressive osseous neoplasm that occurs predominantly during the first two decades of life. Although historically regarded as a reactive process, the identification of recurrent USP6 rearrangements established the neoplastic nature of primary ABCs and serves as a valuable molecular marker in diagnostically challenging cases [1,2]. These lesions most commonly affect the long bones and vertebral column, whereas involvement of the skull base is uncommon [1,3].

The clivus may be affected by a broad spectrum of benign and malignant lesions with overlapping clinical and radiological features, making definitive diagnosis particularly challenging [4]. We report a primary ABC centered in the clivus with extension into the sphenoid sinus and right cavernous sinus. An initial endoscopic transsphenoidal biopsy yielded a histopathological diagnosis that was discordant with the lesion’s progressive clinical and radiological behavior. This prompted a second transcranial microsurgical procedure for repeat tissue sampling, with subsequent histopathological and molecular findings, including a USP6 rearrangement, supporting the diagnosis of primary ABC in the appropriate clinicopathological context. This case highlights the importance of clinicoradiological-pathological correlation, diagnostic reassessment, and ancillary molecular testing when histopathological findings are discordant with the clinical and radiological course.

## Case presentation

A 59-year-old woman initially presented in 2022 with a three-month history of right frontal headache radiating to the ophthalmic (V1) distribution of the trigeminal nerve. Neuroimaging was subsequently performed. Over the course of the disease, she developed progressive binocular diplopia, followed by right-sided ptosis and worsening ophthalmoparesis. Neurological examination demonstrated partial right ptosis, mydriasis, marked limitation of extraocular movements, with the right eye resting in slight adduction, and hypoesthesia in the V1 distribution. Visual acuity and funduscopic examination were preserved.

Imaging findings

**Figure 1:**
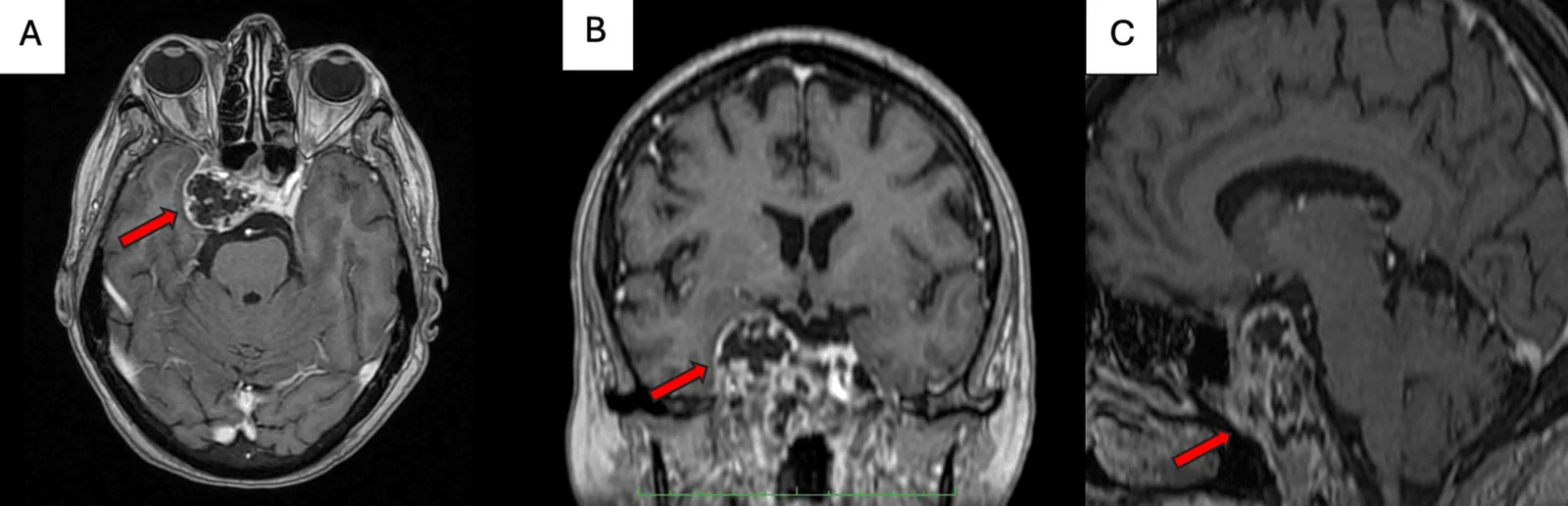
MRI of the clival lesion in October 2024 MRI obtained in October 2024. (A) Axial, (B) coronal, and (C) sagittal contrast-enhanced T1-weighted images demonstrating an expansile heterogeneous lesion centered in the clivus, with extension into the sphenoid sinus and right cavernous sinus. Red arrows indicate the lesion and its extension.

An earlier MRI performed in February 2023 was reported to demonstrate a clival lesion measuring 21×15×10 mm; the original images from this examination were unavailable for retrospective review. MRI performed in October 2024 demonstrated an expansile lesion involving the clivus and right cavernous sinus, extending anteriorly into the sphenoid sinus and measuring approximately 3.9×3.1×2.8 cm (Figure 1).

The lesion demonstrated heterogeneous contrast enhancement and displaced the cavernous segment of the right internal carotid artery laterally. On noncontrast T1-weighted imaging, the lesion demonstrated heterogeneous, predominantly iso- to hypointense signal, whereas on T2-weighted imaging, it was markedly hyperintense and heterogeneous, with a multilobulated internal appearance. No definite fluid-fluid levels were identified. Susceptibility-weighted imaging (SWAN) demonstrated internal foci of susceptibility signal loss, which may reflect blood products and/or mineralization.

**Figure 2:**
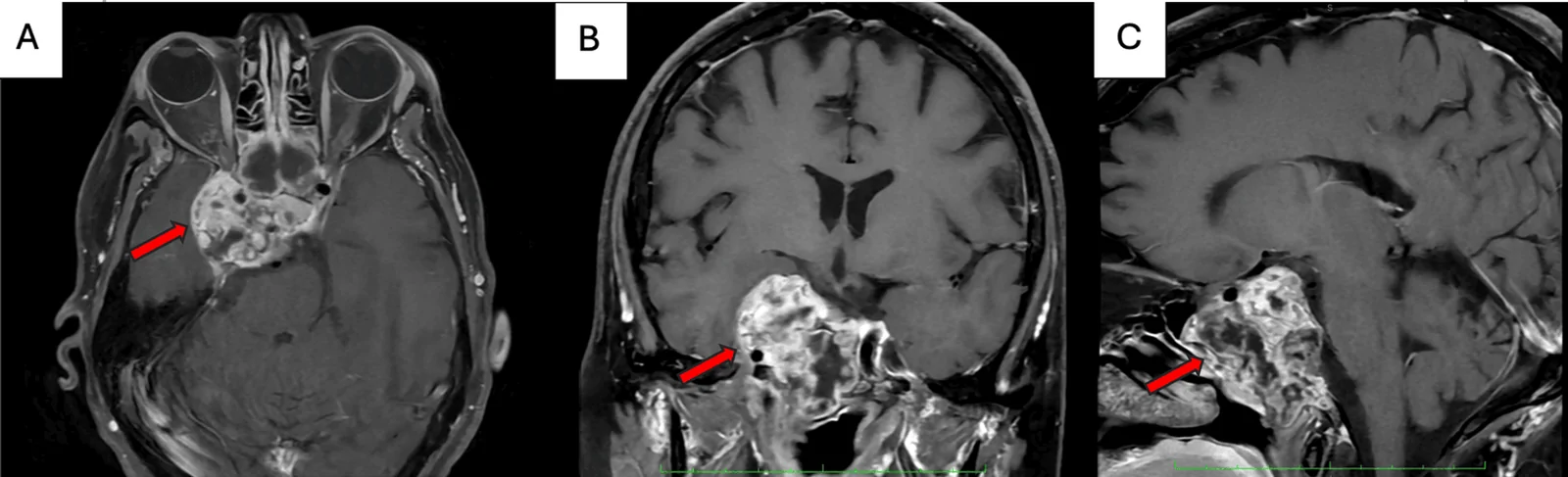
Follow-up MRI demonstrating lesion progression in August 2025 (A) Axial, (B) coronal, and (C) sagittal contrast-enhanced T1-weighted MR images obtained in August 2025 demonstrate a large, heterogeneous, expansile lesion centered in the clivus, with extension into the sphenoid sinus and right cavernous sinus. The lesion measured 5.7×5.1×4.8 cm, compared with 5.6×4.8×4.3 cm on MRI obtained in May 2025. Red arrows indicate the lesion in each plane.

MRI performed in May 2025 demonstrated further enlargement of the lesion to 5.6×4.8×4.3 cm. A subsequent MRI in August 2025, three months later, demonstrated further progression to 5.7×5.1×4.8 cm, with increasing involvement of the skull base and compression of adjacent neurovascular structures (Figure 2).

**Figure 3:**
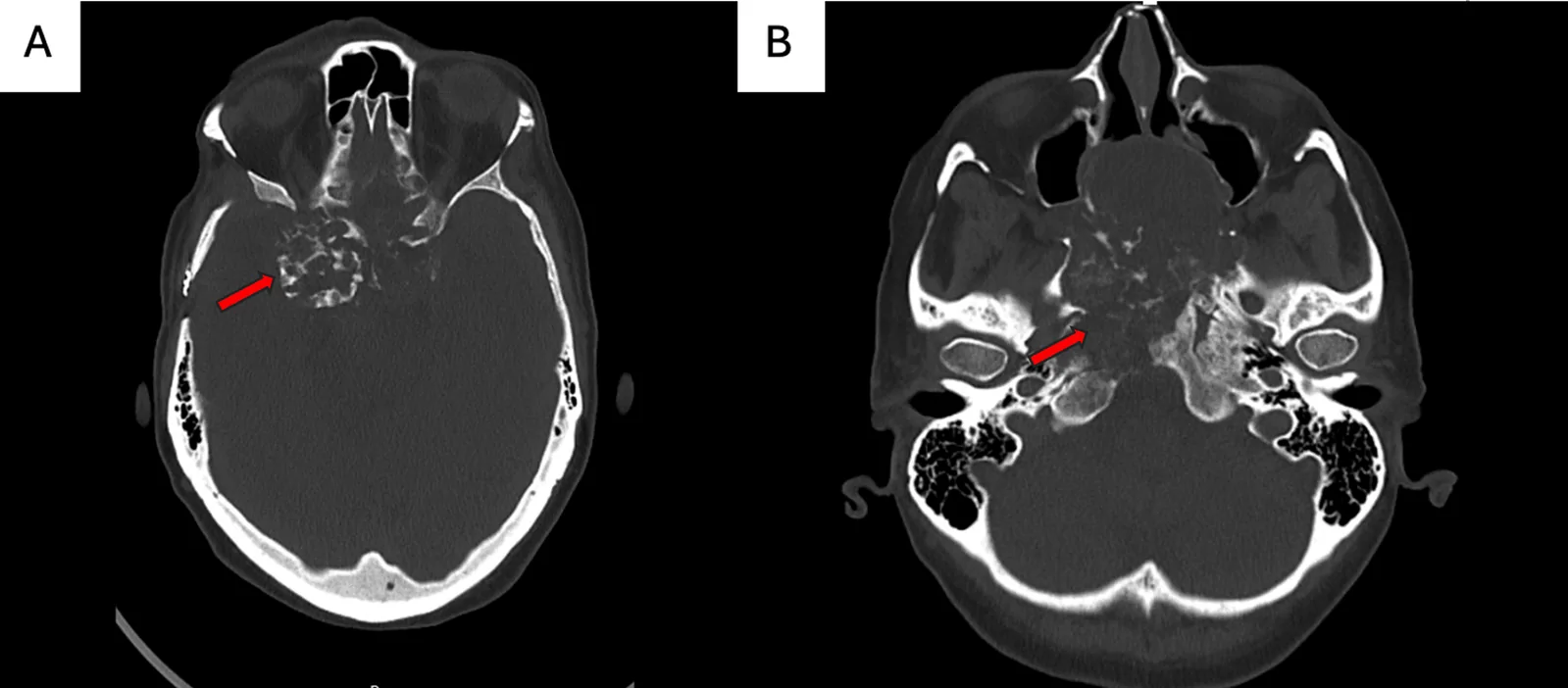
Computed tomography demonstrating osseous destruction of the skull base in May 2025 (A-B) Axial computed tomography images obtained in May 2025 in the bone window demonstrate an expansile, destructive lesion centered in the clivus, with extensive osseous erosion and involvement of the adjacent skull base. Red arrows indicate the areas of osseous involvement.

Computed tomography performed in May 2025, at the time of the second surgical procedure, demonstrated an aggressive expansile osteolytic lesion centered in the clivus, with extensive bone destruction involving the sphenoid sinus, sella turcica, petrous apex, sphenoid wings, and adjacent skull base structures (Figure 3).

Initial diagnostic procedure

In July 2024, considering the lesion’s location and uncertain diagnosis, an endoscopic endonasal transsphenoidal biopsy was performed. No dedicated vascular imaging with computed tomography angiography, magnetic resonance angiography, or digital subtraction angiography was obtained before either procedure, and preoperative embolization was not considered or performed. Intraoperatively, a bulging, brownish lesion was identified within the sphenoid sinus. The lesion appeared highly vascular, and representative biopsy specimens were obtained for histopathological examination.

Histopathological analysis was interpreted as compatible with a mucocele. Fluorescence in situ hybridization (FISH) analysis was not performed on the initial biopsy specimen. Whether the discrepancy between the initial and definitive diagnoses resulted from nonrepresentative sampling or interpretive factors could not be determined retrospectively.

Despite the initial pathological diagnosis, the patient continued to experience progressive headache, worsening ophthalmoplegia, and persistent cranial neuropathies. Serial imaging also demonstrated continued lesion enlargement.

Definitive surgical management

Given the progressive neurological deterioration and continued radiological enlargement despite the initial histopathological diagnosis, the patient underwent a second procedure in May 2025 through a right pterional craniotomy. The lesser wing of the sphenoid was drilled to facilitate exposure of the roof of the cavernous sinus, which was then carefully opened to obtain representative tissue from the lesion. Moderate intraoperative bleeding occurred and was successfully controlled. Owing to the lesion’s intimate relationship with the cavernous sinus, internal carotid artery, and surrounding neurovascular structures, only a limited amount of tissue could be safely removed. No intraoperative or postoperative surgical complications occurred, and no new neurological deficits developed; the pre-existing neurological deficits persisted postoperatively.

Histopathological and molecular findings

**Figure 4:**
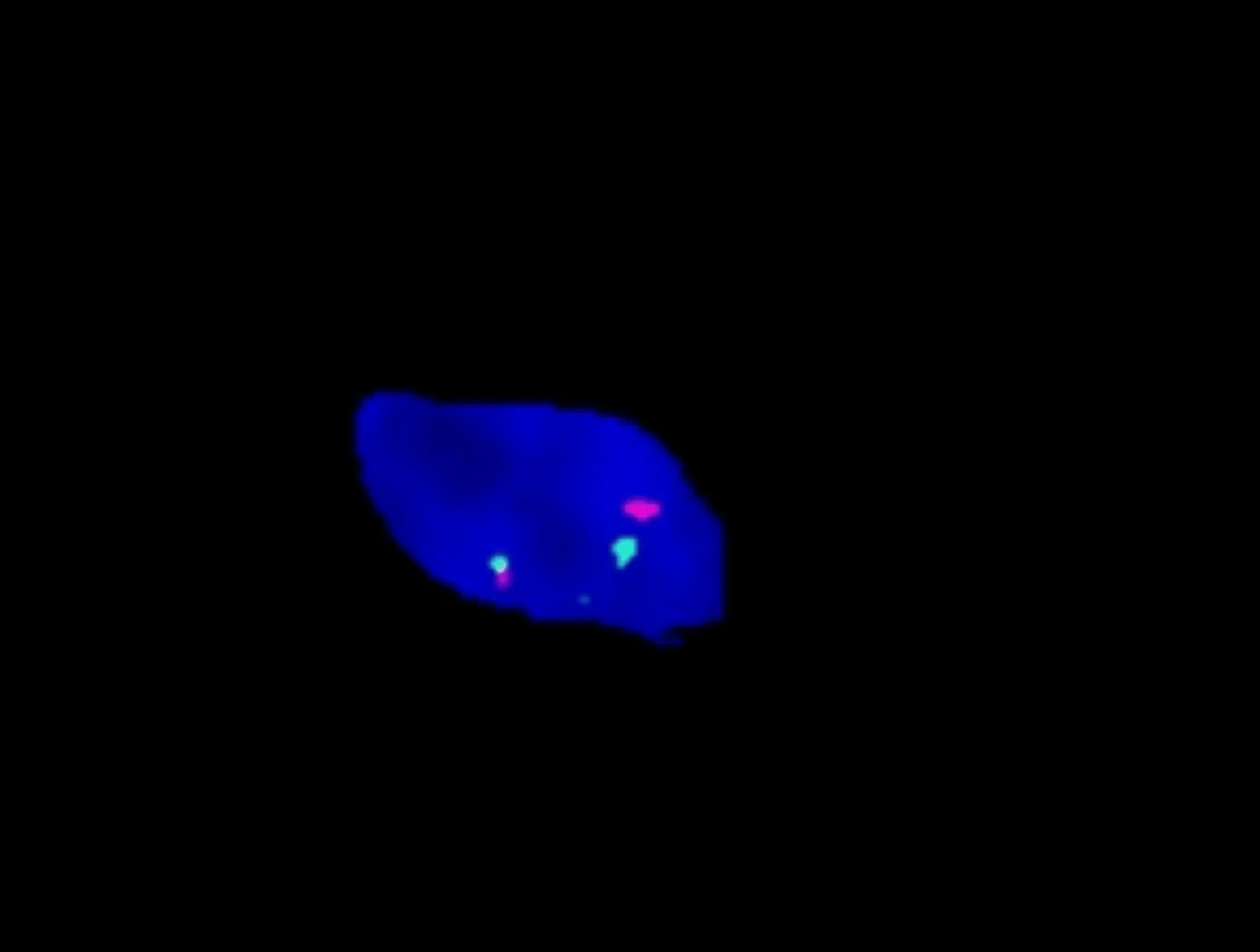
FISH analysis for USP6 Fluorescence in situ hybridization (FISH) analysis was performed using the ZytoLight SPEC USP6 Dual Color Break Apart Probe. An abnormal rearrangement pattern involving the USP6 locus was identified in 20 of 100 evaluated cells. The break-apart assay demonstrated rearrangement of the USP6 locus but did not identify the fusion partner. This finding provided molecular support for the diagnosis of primary aneurysmal bone cyst in the appropriate clinicopathological context.

Histopathological examination of the tissue obtained from the right cavernous sinus demonstrated spindle-cell and osteocartilaginous proliferation. Immunohistochemical analysis showed smooth muscle actin (SMA) positivity in areas of myofibroblastic proliferation, while AE1/AE3, brachyury, and H3K36M were negative. The available pathology report did not specifically document blood-filled cystic spaces, osteoid-containing septa, or multinucleated giant cells. Mitotic activity, cytological atypia, and necrosis were not specifically documented, and the available material did not permit reliable retrospective classification as conventional versus predominantly solid ABC. Given the unusual anatomical location, FISH analysis was performed and demonstrated a USP6 gene rearrangement in 20 of 100 evaluated cells, supporting the diagnosis of primary ABC in the appropriate clinicopathological context (Figure 4).

Clinical outcome

The patient’s pre-existing cranial nerve deficits remained unchanged after surgery. Following the final integrated diagnosis, the patient was referred to clinical oncology for consideration of denosumab because complete surgical excision was not feasible. After multidisciplinary evaluation, denosumab was not initiated because the available evidence supporting its use in ABC was considered limited, consisting predominantly of case reports and small case series. A specific dosing regimen was therefore not established. The lesion continued to progress radiologically and clinically. The patient subsequently developed marked neurological deterioration, with decreased level of consciousness and worsening cranial nerve dysfunction, including bilateral pupillary dilation, in the setting of progressive local disease with probable brainstem compression. She died in March 2026, approximately 10 months after the final diagnosis.

The overall clinical, radiological, diagnostic, and therapeutic course is summarized in Table 1.

**Table 1:** Timeline of the clinical course ABC, aneurysmal bone cyst; FISH, fluorescence in situ hybridization

Time point	Clinical/diagnostic event	Key findings and management
2022	Initial clinical presentation	Right frontal headache radiating to the ophthalmic (V1) distribution of the trigeminal nerve, reported as having been present for approximately three months at initial evaluation.
Feb 23	MRI	Radiology report documented a clival lesion measuring 21×15×10 mm; the original images were unavailable for retrospective review.
Subsequent clinical course	Progressive cranial neuropathies	Development of binocular diplopia, followed by right-sided ptosis, progressive ophthalmoparesis, mydriasis, and V1 hypoesthesia.
Jul 24	Initial endoscopic transsphenoidal biopsy	Endoscopic biopsy of the sphenoid sinus lesion; initial histopathological interpretation was compatible with a mucocele.
Oct 24	MRI	Expansile clival lesion measuring 3.9×3.1×2.8 cm, with extension toward the sphenoid and right cavernous sinus.
May 25	Follow-up MRI and CT	MRI demonstrated enlargement to 5.6×4.8×4.3 cm. CT demonstrated extensive osteolytic destruction of the clivus and adjacent skull base structures.
May 25	Right pterional transcranial procedure	Repeat tissue sampling from the right cavernous sinus; moderate but controlled bleeding; no new postoperative neurological deficits.
May 25	Definitive pathological assessment	Spindle cell and osteocartilaginous proliferation; immunohistochemical findings and USP6 rearrangement by FISH supported the diagnosis of primary ABC in the appropriate clinicopathological context.
Aug 25	Subsequent MRI	Further enlargement to 5.7×5.1×4.8 cm, with progressive involvement of adjacent neurovascular structures.
After definitive diagnosis	Multidisciplinary treatment assessment	Denosumab was considered after referral to clinical oncology but was not recommended because of the limited supporting evidence in this setting.
Subsequent course	Progressive local disease	Neurological deterioration, including decreased level of consciousness and bilateral mydriasis, in the setting of probable brainstem compression.
Mar 26	Final outcome	Patient died following continued clinical and radiological progression.

## Discussion

Primary ABC is a benign but locally aggressive bone neoplasm characterized by recurrent USP6 gene rearrangements, which establish its neoplastic nature and represent an important molecular feature of primary lesions [1,2,5]. Although ABCs predominantly affect the long bones and vertebral column, skull base involvement is distinctly uncommon [1,3]. The age of the present patient was also highly atypical, as ABCs occur predominantly in children and young adults [1,2]. Lesions centered in the clivus present a particular diagnostic challenge because of their proximity to critical neurovascular structures and the broad differential diagnosis of destructive skull base lesions [4,6,7].

The radiological differential diagnosis of clival lesions includes chordoma, chondrosarcoma, giant cell tumor, plasmacytoma, metastatic disease, fibrous dysplasia, and inflammatory lesions such as mucoceles [4,6,7]. Although imaging is essential for lesion characterization and surgical planning, considerable overlap exists among these entities. Classically described imaging features of ABC, including expansile remodeling, internal septations, and fluid-fluid levels, are not universally present and should not be considered pathognomonic [1,6,7]. In the present case, no definite fluid-fluid levels were identified, further illustrating that the absence of this classic feature does not exclude ABC. Consequently, radiological findings should be interpreted in conjunction with the clinical presentation and pathological findings.

A key aspect of the present case was the marked discordance between the initial pathological diagnosis and the subsequent clinical and radiological evolution. The first endoscopic transsphenoidal biopsy was interpreted as a mucocele; however, continued aggressive osteolytic enlargement accompanied by progressive cranial neuropathies raised concern that the initial diagnosis did not adequately explain the lesion’s behavior. Recognition of this discrepancy prompted repeat tissue sampling through a transcranial microsurgical approach, leading to diagnostic reassessment based on the subsequent histopathological, immunohistochemical, and molecular findings. Similar diagnostic difficulties have been reported in ABCs involving the clivus and sphenoid region, where their clinical and radiological appearance may mimic other skull base lesions [4,6,7]. This case therefore highlights the importance of integrating clinical, radiological, and pathological findings and suggests that persistent clinicopathological discordance should prompt diagnostic reassessment rather than continued reliance on an initial biopsy result.

Histopathological interpretation of ABC may be challenging, particularly in unusual anatomical locations and when the differential diagnosis includes other giant cell-rich or cystic bone lesions [1,5]. In the present case, the available pathology report described spindle-cell and osteocartilaginous proliferation but did not specifically document classic morphological features such as blood-filled cystic spaces, osteoid-containing septa, or multinucleated giant cells. Mitotic activity, cytological atypia, and necrosis were also not specifically documented, and representative histopathological photomicrographs were unavailable for retrospective reassessment. Consequently, reliable retrospective classification as conventional versus predominantly solid ABC was not possible. These limitations underscore the importance of interpreting the pathological findings together with the clinical, radiological, and ancillary molecular data.

Identification of USP6 rearrangements has become an important ancillary diagnostic tool in primary ABC [1,2,5,8]. Molecular studies have demonstrated USP6 rearrangements in a substantial proportion of primary ABCs and have shown their absence in secondary ABC-like changes associated with several other bone tumors [1,5,8]. However, USP6 rearrangement should not be interpreted in isolation, as alterations involving USP6 have also been described in other benign mesenchymal lesions [1]. In the present case, the USP6 rearrangement was therefore interpreted as supportive of primary ABC in the appropriate clinicopathological context, rather than independently diagnostic. The break-apart FISH assay demonstrated rearrangement of the USP6 locus but did not identify the fusion partner [2]. Thus, the molecular finding served as an ancillary component of the integrated diagnostic assessment.

Complete surgical excision remains a preferred treatment when it can be achieved safely; however, skull base ABCs may involve critical neurovascular structures and may be associated with substantial intraoperative bleeding, limiting the feasibility of radical resection without unacceptable morbidity [9,10]. Consequently, management should be individualized according to lesion location, neurological status, and operative risk. Preoperative selective arterial embolization has been used in skull base ABCs and may reduce intraoperative blood loss [9]. In the present case, no dedicated preoperative vascular study was performed, and embolization was not considered. More broadly, several treatment strategies have been described for primary ABCs, although comparative evidence remains limited [11].

Denosumab has emerged as a potential therapeutic option for ABCs in which conventional surgical management is difficult or associated with substantial morbidity [12-14]. Its use has also been reported specifically in a massive ABC of the skull base [12]. Nevertheless, the available evidence remains predominantly observational, and uncertainties persist regarding optimal treatment duration, recurrence after discontinuation, and potential metabolic adverse effects [14]. In our patient, complete surgical excision was not considered feasible because of the extensive skull base and cavernous sinus involvement. The patient was referred to clinical oncology for consideration of denosumab; however, the oncology team did not recommend treatment because the available evidence supporting its use in this setting was considered insufficient. The lesion subsequently continued to progress, with neurological deterioration and an ultimately unfavorable outcome.

Previous reports further illustrate the diagnostic and therapeutic heterogeneity of skull base ABCs. Asi et al. described a massive skull base ABC treated with denosumab [12], whereas a recently reported solid clival ABC in a 67-year-old patient demonstrated that these lesions may also occur in older adults and mimic other skull base neoplasms [15]. In contrast, the present case was characterized by longitudinally documented progression, an initially discordant pathological diagnosis, progressive cavernous sinus involvement, and subsequent detection of a USP6 rearrangement supporting diagnostic reassessment. The markedly unfavorable clinical course in our patient further contrasts with the favorable immediate postoperative outcome reported in the latter case [15].

This case has limitations inherent to its retrospective nature. Representative histopathological photomicrographs were unavailable for retrospective review, limiting further morphological characterization of the lesion. In addition, the original images from the February 2023 MRI were unavailable, and measurements from this examination were therefore based on the contemporaneous radiology report.

## Conclusions

ABC should be considered in the differential diagnosis of destructive clival lesions, even in older adults and in the absence of classic radiological features. This case illustrates the diagnostic difficulty posed by ABCs in unusual skull base locations and emphasizes the importance of reassessing the diagnosis when histopathological findings are discordant with progressive clinical and radiological behavior. In such cases, repeat tissue sampling and ancillary molecular testing for USP6 rearrangement may provide valuable diagnostic support when interpreted in the appropriate clinicopathological context. Extensive involvement of the cavernous sinus and adjacent neurovascular structures may substantially limit surgical treatment, underscoring the challenges associated with locally aggressive skull base ABCs.
